# Nomogram for predicting the surgical difficulty of laparoscopic total mesorectal excision and exploring the technical advantages of robotic surgery

**DOI:** 10.3389/fonc.2024.1303686

**Published:** 2024-01-26

**Authors:** Fangliang Guo, Cong Xia, Zongheng Wang, Ruiqi Wang, Jianfeng Gao, Yue Meng, Jiahao Pan, Qianshi Zhang, Shuangyi Ren

**Affiliations:** ^1^ Department of Gastrointestinal Surgery, The Second Affiliated Hospital of Dalian Medical University, Dalian, Liaoning, China; ^2^ Department of Public Health, China Medical University, Shenyang, Liaoning, China; ^3^ Department of General Surgery, Shanghai Changzheng Hospital, Shanghai, China

**Keywords:** total mesorectal excision, surgical difficulty, laparoscopic surgery, robotic surgery, technically challenging rectal cancer

## Abstract

**Background:**

Total mesorectal excision (TME), represents a key technique in radical surgery for rectal cancer. This study aimed to construct a preoperative nomogram for predicting the surgical difficulty of laparoscopic total mesorectal excision (L-TME) and to investigate whether there were potential benefits of robotic TME (R-TME) for patients with technically challenging rectal cancer.

**Methods:**

Consecutive mid­low rectal cancer patients receiving total mesorectal excision were included. A preoperative nomogram to predict the surgical difficulty of L-TME was established and validated. Patients with technically challenging rectal cancer were screened by calculating the prediction score of the nomogram. Then patients with technically challenging rectal cancer who underwent different types of surgery, R-TME or L-TME, were analyzed for comparison.

**Results:**

A total of 533 consecutive patients with mid­low rectal cancer who underwent TME at a single tertiary medical center between January 2018 and January 2021 were retrospectively enrolled. Multivariable analysis demonstrated that mesorectal fat area, intertuberous distance, tumor size, and tumor height were independent risk factors for surgical difficulty. Subsequently, these variables were used to construct the nomogram model to predict the surgical difficulty of L-TME. The area under the receiver operating characteristic curve of the nomogram was 0.827 (95% CI 0.745 - 0.909) and 0.809 (95% CI 0.674- 0.944) in the training and validation cohort, respectively. For patients with technically challenging rectal cancer, R-TME was associated with a lower diverting ileostomy rate (p = 0.003), less estimated blood loss (p < 0.043), shorter procedure time (p = 0.009) and shorter postoperative hospital stay (p = 0.037).

**Conclusion:**

In this study, we established a preoperative nomogram to predict the surgical difficulty of L-TME. Furthermore, this study also indicated that R-TME has potential technical advantages for patients with technically challenging rectal cancer.

## 1 Introduction

Colorectal cancer is the third most common malignant tumor worldwide. Rectal cancer, with increasing incidence and mortality rates, has become the most common malignant tumor of the digestive system in the last decades (1, 2). Environmental factors, dietary habits, physical activity, and hereditary factors are the main factors contributing to the development of rectal cancer. Although treatment strategies include surgical resection, radiation therapy, chemotherapy, targeted therapy, and immunotherapy, surgical treatment remains the mainstay of curative treatment for rectal cancer (3). Total mesorectal excision (TME) is the standard procedure for mid-low rectal cancer surgery (4). With surgeons’ talents and efforts, laparoscopic TME (L-TME), whose advantages have been extensively reported in previous studies versus open surgery, is widely applied in clinics (5, 6). Nevertheless, L-TME is a technically demanding procedure that requires skillful dissection of the tumor-bearing rectum and its surrounding mesentery in the pelvis. Specifically, operating in the low pelvis with conventional straight instruments is extremely challenging in patients with technically challenging rectal cancer (7).

In contrast, robotic TME (R-TME), a cutting-edge surgical technique, has emerged as a promising option for overcoming the surgical difficulty associated with L-TME (8–13). Despite some controversy about the universal benefits of R-TME, the ROLARR trial suggested that R-TME may offer potential advantages for technically challenging patients (12). In addition, several studies have also reported that R-TME allows surgeons to feel comfortable and achieve better results when performing technically demanding operations, such as those with a narrow pelvis or low-level tumor (13–15). However, considering the additional financial burden and time overhead of robotic surgery, it is important to screen patients who could benefit from robotic surgery.

In this context, we constructed a preoperative nomogram to predict the surgical difficulty of L-TME and investigated whether there were potential benefits of R-TME for patients with technically challenging rectal cancer.

## 2 Materials and methods

### 2.1 Patients

**Figure 1:**
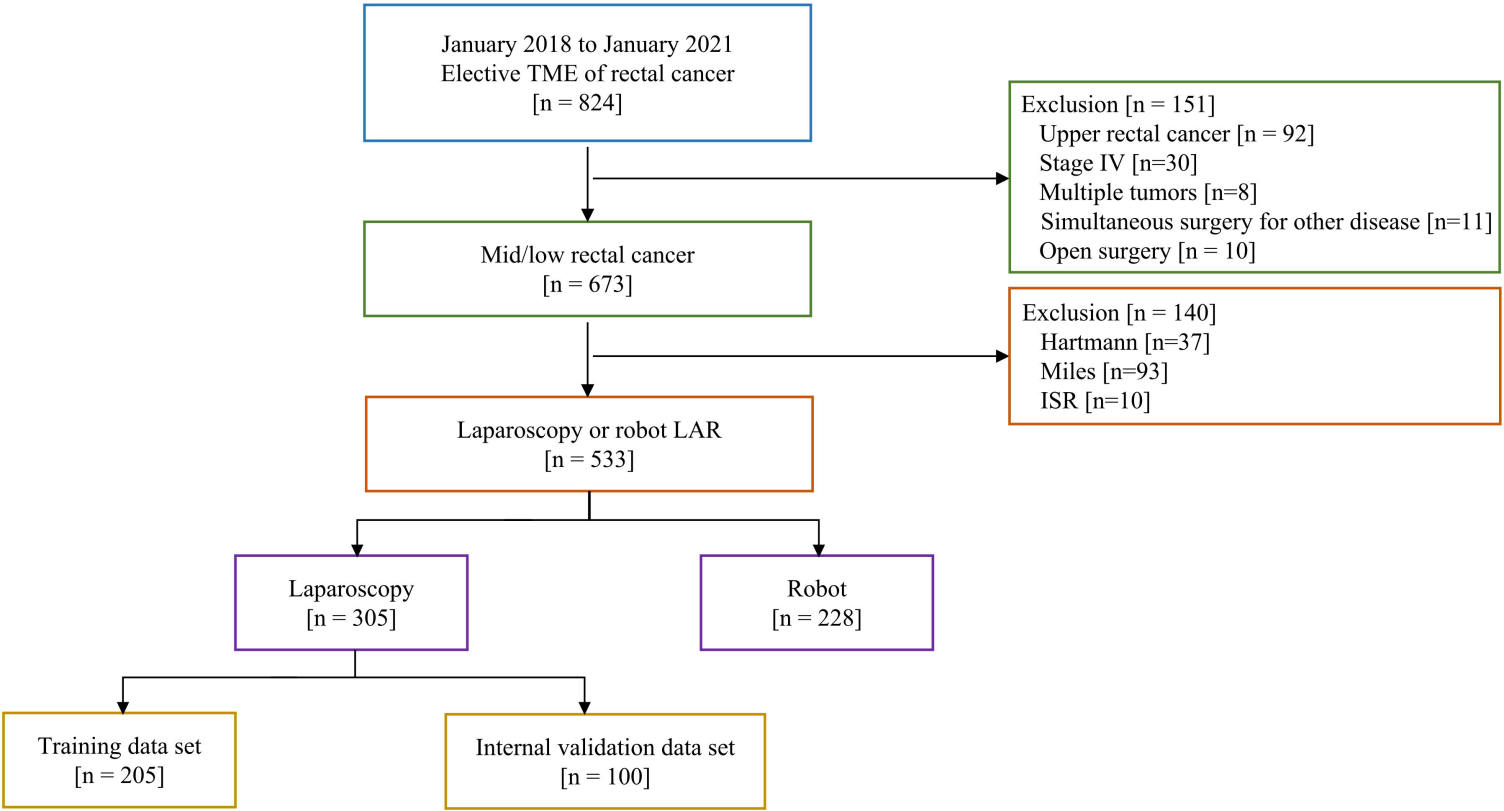
Flowchart of the patients assessed in this study.

Data of consecutive mid­low rectal cancer patients who underwent elective TME between January 2018 and January 2021, were retrospectively retrieved from our prospectively collected database. The inclusion criteria were as follows: (1) pathologic diagnosis of adenocarcinoma of the colorectum; (2) elective radical operation; (3) age ≥ 18 years. The exclusion criteria included: (1) stage IV; (2) upper rectal cancer (distance >10 cm from the anus); (3) multiple tumors; (4) simultaneous surgery for other diseases; (5) intersphincteric resection (ISR), abdominoperineal resection (APR) or Hartmann’s procedure. The flowchart of the patient selection is shown in **Figure 1**.

### 2.2 Surgical procedure

The surgical techniques were performed as described in previous reports (10, 16). In brief, the rectum and mesentery were dissected and mobilized according to the principle of TME and the medial-to-lateral approach. After the intracorporeal rectal transection, reconstruction was accomplished using a circular stapler. Following the anastomosis, a diverting ileostomy was selectively created at a distance of approximately 20 cm from the oral side of the terminal ileum. All procedures were performed by a surgeon (Shuangyi Ren) with experience performing more than 2000 laparoscopic surgeries (16, 17).

### 2.3 Variable and outcome definition

**Figure 2:**
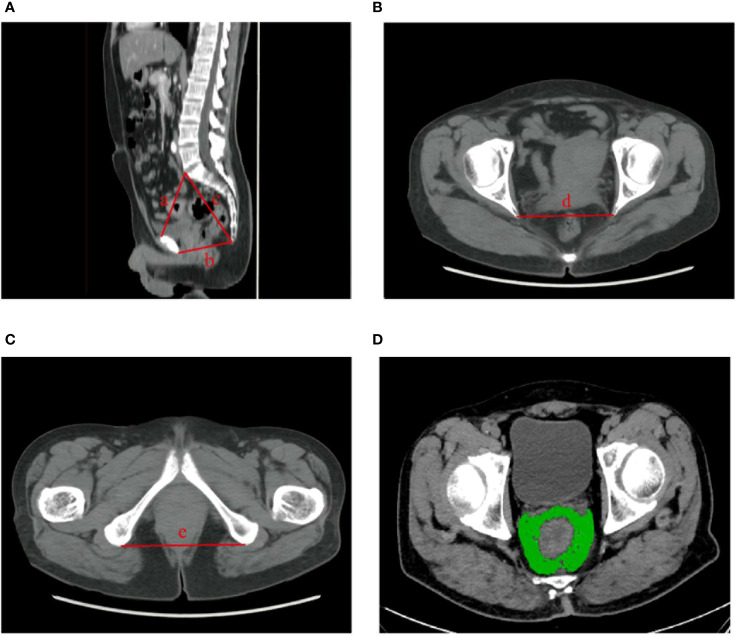
Measurements of pelvimetric parameters. **(A)** pelvic inlet (line a); pelvic outlet (line b); pelvic depth (line b). **(B)** interspinous distance (line d). **(C)** intertuberous distance (line e). **(D)** mesorectal fat area (depicted in green).

Pelvimetry parameters were measured on lateral and axial computed tomography (CT) images as described previously (18). Pelvic inlet was defined as distance between promontory and the superior edge of symphysis pubis. Pelvic depth was the distance between promontory and coccyx. Pelvic outlet was the distance between coccyx and the inferior edge of the symphysis pubis. Interspinous distance corresponded to the transverse distance between the tips of ischial spines. Intertuberous distance corresponded to the transverse distance between the lowest points of ischial tuberositie. Additionally, the mesorectal fat area (MFA) was measured at the level of the tip of the sciatic spine using Slice-O-matic software, version 4.3 (Tomovision, Montreal, QC, Canada) (19) (**Figure 2**).

The surgical difficulty criteria were referred to the definition previously given by Escal et al. (18) with modifications: operation time > 180 min (3 points), conversion to open surgery (3 points), transanal approach (2 points), postoperative hospital stay > 7 days (2 points), estimated blood loss ≥ 100 ml (1 points), Clavien–Dindo classifications grade II and III postoperative morbidities (1 point). Based on the surgical difficulty score, patients were classified into two subgroups, difficulty (≥ 6 points) and non-difficulty (0–5 points) groups (**Table 1**).

**Table 1:** Surgical difficulty grading.

	Points
Operation time > 180 min	3
Conversion to open surgery	3
Transanal approach	2
Postoperative hospital stay > 7 days	2
Estimated blood loss ≥ 100 ml	1
Morbidity (grade II - III)	1

### 2.4 Construction and validation of the nomogram

Patients who underwent L-TME were divided into two groups. Those who had surgery before January 2020 were included in the training group, while the remaining patients were included in the validation group.

In the training group, univariate logistic regression analysis was performed to initially assess the associations of various indexes with surgical difficulty. All indexes with a p-value < 0.1 were included in the multivariate logistic analysis. R software (version 4.2.1; http://www.r-project.org/) was used to construct a nomogram based on multivariate analysis. Receiver operating characteristic (ROC) curves and calibration curves were used to evaluate the discrimination and accuracy, respectively. The nomogram-based point of each patient was calculated by the “nomogramFormula” package.

### 2.5 Statistical analysis

SPSS 25.0 (IBM Corp, Armonk, New York, USA) was used for statistical analyses. Continuous data were displayed as mean (± standard deviation [SD]) or median (interquartile range [IQR]) while categorical data were displayed as n (%). Student’s t-test or Wilcoxon rank-sum test was used for continuous variables (ANOVA and Kruskal–Wallis test for multi-component data), and chi-squared test or Fisher’s exact test was used for categorical variables. All statistical tests were two-sided and statistical significance was set at p-value < 0.05.

## 3 Results

### 3.1 Patient characteristics

A total of 814 patients were reviewed, of which 533 were included in our study. There were 305 laparoscopic and 228 robotic cases. Based on the time of surgery, patients undergoing L-TME were divided into a training group (n = 205, 67.2%) and a validation group (n = 100, 32.8%). The baseline and imaging characteristics of the patients are presented in **Tables 2** and **3**. As expected, there were no statistical differences in these characteristics among the laparoscopic training group, laparoscopic validation group, and robotic group.

**Table 2:** Baseline characteristics of patients undergoing TME.

	Laparoscopy training group (N = 205)	Laparoscopy internal validation group (N = 100)	Robot group (N = 228)	p
Sex [n (%)]				0.613
Male	125 (61.0%)	65 (65.0%)	135 (59.2%)	
Female	80 (39.0%)	35 (35.0%)	93 (40.8%)	
Age [median (IQR), years]	64 (59-70)	66 (66-71.8)	63.5 (57-70)	0.319
Hypertension [n (%)]	55 (26.8%)	26 (26.0%)	56 (24.6%)	0.862
Diabetes [n (%)]	28 (13.7%)	13 (13.0%)	22 (9.6%)	0.401
Cardiac disease [n (%)]	10 (4.9%)	5 (5.0%)	18 (7.9%)	0.369
Respiratory disease [n (%)]	17 (8.3%)	12 (12.0%)	23 (10.1%)	0.577
ASA score [n (%)]				0.055
I	121 (59.0%)	45 (45.0%)	136 (59.7%)	
II	73 (35.6%)	48 (48.0%)	73 (32.0%)	
III	11 (5.4%)	7 (7.0%)	19 (8.3%)	
Smoking history [n (%)]	57 (27.8%)	31 (31.0%)	52 (22.8%)	0.245
Drinking history [n (%)]	29 (14.1%)	13 (13.0%)	27 (11.8%)	0.775
Preoperative chemotherapy [n (%)]	15 (7.3%)	10 (10%)	17 (7.5%)	0.682
Preoperative radiotherapy [n (%)]	12 (5.9%)	9 (9.0%)	12 (5.3%)	0.420
Previous abdominal surgery [n (%)]	22 (10.7%)	16 (16.0%)	37 (16.2%)	0.215
CEA abnormal [n (%)]	49 (23.9%)	18 (18.0%)	49 (21.5%)	0.498
CA 199 abnormal [n (%)]	9 (4.4%)	4 (4.0%)	10 (4.4%)	0.985
Anemia [n (%)]	24 (11.7%)	13 (13.0%)	26 (11.4%)	0.917
Hypoproteinemia [n (%)]	19 (9.3%)	7 (7.0%)	19 (8.3%)	0.797
Tumor size [median (IQR), cm]	4 (3-5)	4 (3-5)	4.5 (3.5-5.5)	0.438
Tumor height [median (IQR), cm]	8 (6-10)	8 (5-10)	7.9 (5-10)	0.490
Tumor differentiation [n (%)]				
Poor	22 (10.7%)	9 (9.0%)	32 (14.0%)	0.191
Moderate	162 (79%)	81 (81.0%)	161 (70.6%)	
High	21 (10.2%)	10 (10.0%)	35 (15.4%)	
Pathological T stage [n (%)]				0.809
T1	23 (11.2%)	13 (13.0%)	23 (10.0%)	
T2	46 (22.4%)	20 (20.0%)	49 (21.5%)	
T3	110 (53.7%)	48 (48.0%)	121 (53.1%)	
T4	26 (12.7%)	19 (19.0%)	35 (15.4%)	
Pathological N stage [n (%)]				0.621
N0	138 (67.3%)	60 (60%)	142 (62.3%)	
N1	38 (18.5%)	23 (23.0%)	44 (19.3%)	
N2	29 (14.1%)	17 (17.0%)	42 (18.4%)	
Tumor stage [n (%)]				0.142
I	46 (22.4%)	29 (29.0%)	46 (20.2%)	
II	92 (44.9%)	31 (31.0%)	96 (42.1%)	
III	67 (32.7%)	40 (40%)	86 (37.7%)	
BMI [mean (SD), kg/m²]	24.4± 3.5	24.1± 3.0	24.7± 3.4	0.300

TME, Total mesorectal excision; ASA, American Society of Anesthesiologists Classification; BMI, body mass index; CEA, Carcinoembryonic antigen; CA 199, Carbohydrate antigen 199; IQR, interquartile range; SD, standard deviation.

**Table 3:** Imaging characteristics of patients undergoing TME.

	Laparoscopy training group (N = 205)	Laparoscopy internal validation group (N = 100)	Robot group (N = 228)	p
MFA [mean ± SD, cm^2^]	17.8± 8.5	17.5± 6.3	18.7± 7.7	0.337
Interspinous distance [median (IQR), cm]	99.8 (92.7-109.1)	100.1 (90.3-108.0)	98.8 (92.3-109.2)	0.798
Intertuberous distance [median (IQR), cm]	115.3 (108.2-127.3)	115.3 (106.4-127.1)	117.6 (108.0-128.6)	0.548
Pelvic inlet [median (IQR), cm]	118.8 (111.2-128.5)	120.2 (114.0-128.2)	118.2 (111.7-125.6)	0.522
Pelvic outlet [median (IQR), cm]	93.7 (87.3-99.8)	93.7 (84.6-100.6)	93.6 (87.4-100.6)	0.844
Pelvic depth [median (IQR), cm]	125.1 (115.5-121.3)	124.2 (116.3-134.8)	122.2 (112.0-133.2)	0.311

TME, Total mesorectal excision; MFA, rectal mesenteric fat; IQR, interquartile range; SD, standard deviation.

### 3.2 Development and validation of a predictive nomogram for technically challenging rectal cancer

Multivariate analysis showed that MFA (OR 1.102, 95% CI 1.034-1.174, p = 0.003), intertuberous distance (OR 0.946, 95% CI 0.904-0.989, p = 0.015), tumor height (OR 0.704, 95% CI 0.562-0.883, p = 0.002) and tumor size (OR 1.493, 95% CI 1.087-2.051, p = 0.013) were independent predictive factors of high surgical difficulty of L-TME (**Table 4**).

**Table 4:** Logistic regression analysis of predictors associated with the surgical difficulty of L-TME.

Variables	Univariable analysis		Multivariable analysis	
	OR (95% CI)	P value	OR (95% CI)	P value
Sex (male vs. female)	0.797 (0.350-1.815)	0.589		
Age	1.030 (0.987-1.076)	0.172		
Hypertension	1.532 (0.663-3.539)	0.318		
Diabetes	0.427 (0.096-1.907)	0.265		
Cardiac disease	1.556 (0.313-7.719)	0.589		
Respiratory disease	0.357 (0.046-2.802)	0.327		
ASA score (I/II vs. III)	1.374 (0.282-6.708)	0.694		
Drinking history	1.319 (0.459-3.791)	0.607		
Smoking history	1.200 (0.511-2.818)	0.676		
Preoperative chemotherapy	1.012 (0.215-4.776)	0.988		
Preoperative radiotherapy	3.360 (0.942-11.984)	0.062	3.601 (0.753-17.2223	0.109
Previous abdominal surgery	2.609 (0.927-7.344)	0.069	3.375 (0.932-12.214)	0.064
Anemia	0.852 (0.237-3.060)	0.806		
Hypoproteinemia	1.717 (0.527-5.593)	0.369		
CEA abnormal	2.324 (1.026-5.264)	0.616		
CA19-9 abnormal	1.788 (0.353-9.065)	0.483		
Tumor size	1.517 (1.159-1.985)	0.002	1.493 (1.087-2.051)	0.013
BMI	1.108 (0.990-1.241)	0.074	1.096 (0.940-1.278)	0.243
Tumor differentiation	0.701 (0.297-1.652)	0.416		
Tumor height	0.790 (0.658-0.949)	0.012	0.704 (0.562-0.883)	0.002
Pathological T stage	1.152 (0.707-1.880)	0.570		
Pathological N stage	1.023(0.605-1.759)	0.908		
MFA	1.096 (1.042-1.152)	<0.001	1.102 (1.034-1.174)	0.003
Interspinous distance	0.972 (0.938-1.007)	0.12		
Intertuberous distance	0.954 (0.923-0.985)	0.004	0.946 (0.904-0.989)	0.015
Pelvic inlet	0.970 (0.938-1.004)	0.085	0.979 (0.930-1.029)	0.398
Pelvic outlet	1.003 (0.966-1.041)	0.518		
Pelvic depth	1.034 (1.002-1.068)	0.038	1.032 (0.988-1.079)	0.151

ASA, American Society of Anesthesiologists Classification; BMI, body mass index; CEA, Carcinoembryonic antigen; CA19-9, Carbohydrate antigen 199; MFA, rectal mesenteric fat; CI, Confidence interval.

**Figure 3:**
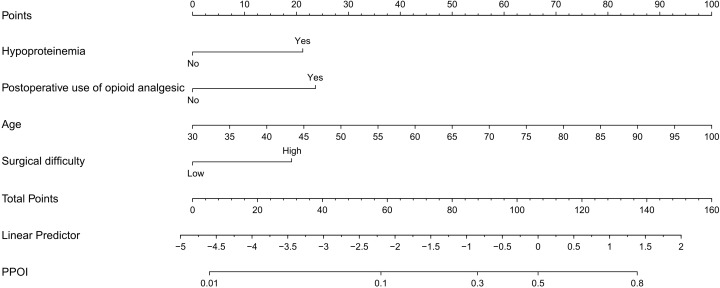
Predictive model of a surgical difficulty nomogram.

**Figure 4:**
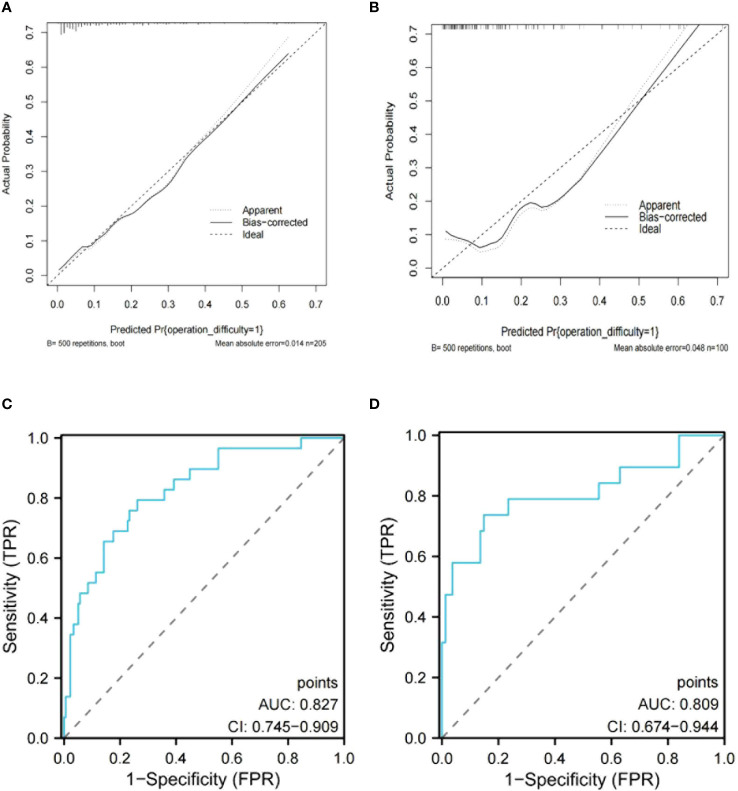
Calibration curve of the nomogram model for training data set **(A)** and internal validation data set **(B)**. The ROC curve of the nomogram model in the training data set **(C)** and internal validation data set **(D)**.

The nomogram model was established using these four variables to assess the surgical difficulty of L-TME (**Figure 3**). The area under the ROC curve (AUC) of the prediction model was 0.827 (95% CI 0.745 - 0.909) for the training dataset and 0.809 (95% CI 0.674- 0.944) for the internal validation dataset. Additionally, the nomogram calibration curve showed acceptable agreement between prediction and actual observation (**Figure 4**).

Patients were calculated by the nomogram and 167 points (the upper quartile) were selected as the cutoff value. The risk stratification was developed (points ≤ 167 as low risk and points > 167 as high risk). Additionally, cases with high risk were defined as technically challenging rectal cancer. The patient features between R-TME and L-TME in patients with technically challenging rectal cancer are summarized in **Supplementary Table S1**.

### 3.3 Comparison between R-TME and L-TME in patients with technically challenging rectal cancer

The subgroup analysis of technically challenging rectal cancer between R-TME and L-TME is shown in **Table 5**. Compared to L-TME, R-TME exhibited a lower rate of diverting ileostomy (p = 0.003), lower estimated blood loss (p = 0.0.43), and shorter postoperative hospital stay (p = 0.037). and higher inpatient cost (p < 0.001). While there was no significant difference between the two groups in terms of operation time (p = 0.738), the procedure time was found to be shorter in the R-TME group after subtracting the docking time (p = 0.009).

**Table 5:** Comparison between R-TME and L-TME in patients with technically challenging rectal cancer.

	L-TME (N = 78)	R-TME (N = 57)	P
Operation time [median (IQR), min]	180 (150-210)	180 (145-210)	0.738
Docking time [median (IQR), min]	4 (3-4)	28 (25-31)	< 0.001
Procedure time [mean ± SD, min]	175 (145.8-206.3)	152 (117-181.5)	0.009
Estimated blood loss [median (IQR), ml]	80 (50-110)	60 (40-105)	0.043
Conversion [n (%)]	3 (3.8%)	2 (3.5%)	1*
Transanal approach [n (%)]	6 (7.7%)	1 (1.8%)	0.238*
Diverting ileostomy [n (%)]	53 (67.9%)	24 (42.1%)	0.003
Time to first flatus [median (IQR), days]	2 (2-3)	2 (1.5-2)	0.055
Time to first stool [median (IQR), days]	3 (2-4)	3 (2-3)	0.434
Time to remove urinary catheter [median (IQR), days]	1 (1-4)	1 (1-3)	0.258
VAS at POD 1 [median (IQR)]	3 (3-4)	3 (3-4)	0.866
VAS at POD 3 [median (IQR)]	2 (2-3)	2 (2-3)	0.665
Distal margin distance [median (IQR), cm]	2 (1-3)	2 (1.2-3)	0.779
Proximal margin distance [median (IQR), cm]	10.5 (9.5-11.1)	10.6 (9.8-11.4)	0.243
Harvested lymph nodes [mean (SD)]	15 (10-23)	16 (10-24)	0.85
CRM+ [n (%)]	4 (5.1%)	1 (1.8%)	0.396
Postoperative complications [n (%)]			0.573*
Grade I	12 (15.4%)	8 (14.0%)	
Grade II	8 (10.3%)	3 (5.3%)	
Grade III	9 (11.5%)	4 (7.0%)	
Grade IV	1 (1.3%)	0	
Surgical complications [n (%)]	22 (28.2%)	9 (18.5%)	0.09
Anastomotic leak	12 (15.4%)	5 (8.8%)	0.253
Wound infection	5 (6.4%)	1 (1.8%)	0.401*
Abdominal abscess	3 (3.8%)	2 (3.5%)	1*
Intraabdominal bleeding	2 (2.6%)	1 (1.8%)	1*
Bowel obstruction	3 (3.8%)	0	0.263*
General complications [n (%)]	15 (19.2%)	6 (10.5%)	0.168
Cardiac complications	5 (6.4%)	1 (1.8%)	0.195*
Pulmonary complications	3 (3.8%)	2 (3.5%)	1*
Deep vein thrombosis	2 (2.6%)	1 (1.8%)	1*
Urinary retention or infection	6 (7.7%)	3 (5.3%)	0.733*
Inpatient cost [median (IQR), $]	9965.6 (9105.2-11156.7)	13028.5 (11970.6-13957.9)	< 0.001
Postoperative hospital stay [median (IQR), days]	8 (6-11)	7 (5.5-9)	0.037
30-day readmission [n (%)]	6 (7.7%)	3 (5.3%)	0.733*
30-day mortality [n (%)]	0	0	–

VAS, visual analog scale; POD, postoperative day; CRM, circumferential radial margin; IQR, interquartile range; SD, standard deviation.

* Using Fisher’s exact test.

Additionally, the robotic group exhibited a lower rate of surgical complications (p = 0.090) and a trend towards faster time to flatus (p = 0.055), although these differences did not reach statistical significance. In terms of surgical specimens, the number of harvested lymph nodes (p = 0.850), distal margin distance (p = 0.779), proximal margin distance (p = 0.243), and positive circumferential margins (p = 0.396) were similar between the two groups.

## 4 Discussion

Rectal cancer accounts for the largest proportion of colorectal cancer in China (20). Its treatment regimens are complex such as neoadjuvant chemoradiotherapy, targeted therapy, and immunotherapy (21, 22). At present, surgical treatment is still the most important treatment for rectal cancer. L-TME is a common surgical approach for rectal cancer (5, 6). However, it still presents challenges, particularly in patients with technically challenging rectal cancer. On the one hand, the rectum is located in the deep part of the pelvis, surrounded by complex tissues, which not only limits maneuverable space but also results in a suboptimal surgical field. On the other hand, this technique has some technical limitations, such as non-articulating instruments, decreased tactile feedback, challenges in achieving optimal anastomotic cutting angles, and an elevated risk of inadvertent collisions (23).

In recent years, more research has focused on nomograms (24). Surgical difficulty prediction nomogram, offering valuable guidance on individualized and precise plans for treatment, such as more sufficient preoperative preparation, more reliable surgical techniques, and more thoughtful postoperative management, are of broad interest to surgeons. Yuan et al. (25) developed a nomogram to predict the difficulty of rectal surgery. However, it’s worth noting that this nomogram model did not include MFA, which has been reported to be an important factor in the difficulty of rectal surgery (26).

In the present study, the clinical and anatomical factors affecting the difficulty of L-TME were analyzed, and a nomogram for predicting the difficulty of L-TME was established and validated, which included intertuberous distance, tumor height from anal verge, rectal mesenteric area, and tumor size.

The effect of pelvic anatomy on the difficulty of rectal resection has been widely reported (18, 27, 28). A narrow pelvis could hinder visibility, and the available workspace during surgery (28). Consistent with previous studies (18, 29), our result reconfirmed that shorter intertuberous distance could represent an anatomical bottleneck of the deep pelvis that hinders operation during L-TME. Larger tumors and lower tumor height can also increase the difficulty of the procedure, making it difficult to transect and anastomose the rectum (27). Cai et al. reported that large tumor size increases the number of linear staplers used during the double stapling technique, which increases the risk of anastomotic leakage (30). Additionally, in patients with large MFA, the space between the mesorectal fascia and the surrounding pelvic fascia will be narrow, which may increase the difficulty of TME (18, 26).

These risk factors are difficult to modify, and further evidence is required to assist surgeons in selecting the most suitable surgical approach for individual patients, which may improve the prognosis for these patients. Compared to laparoscopy, the robot has multiple flexible arms that can perform some operations that are difficult to perform with traditional laparoscopy. Additionally, the robot offers a stable 3D view of the surgical field and digitally suppresses physiological hand tremors (13). These features enable surgeons to perform delicate surgical procedures, even in deep and narrow pelvises. Reportedly, the robotic system provides surgeons with increased comfort and has the potential to overcome the challenges associated with L-TME (13, 31, 32).

In this study, no significant difference was found between R-TME and L-TME in terms of postoperative complications, surgical specimens, and recovery of bowel function in patients with technically challenging rectal cancer. However, we observed that R-TME was associated with shorter hospital stays, which is consistent with previous studies (11).

Interestingly, in this study, the diverting ileostomy rate was lower in the R-TME group than in the L-TME group. Robotic provides surgeons with an improved view and greater flexibility in maneuvering within the pelvic cavity, potentially reducing unexpected trauma to the bowel wall. Robotic surgery allows surgeons to place a reinforced suture at the anastomosis (especially for the so-called dog-ear area) after reconstruction, which is difficult for conventional laparoscopic surgery (33). The lower rate of diverted ileostomy reflects surgeon confidence in robotic anastomosis.

In terms of operative time, it is generally accepted that robotic surgery tends to take longer than laparoscopic surgery (34). Interestingly, in this study, the operation time was similar between R-TME and L-TME for patients with technically challenging rectal cancer. Following the deduction of docking time, the procedure time for R-TME was even shorter than that of L-TME. In addition, the estimated blood loss was lower in R-TME.

This may be due to the fact that technically challenging rectal cancer is often associated with a narrow surgical field and a crowded operating space, which requires significant technical expertise to perform precise operations. When L-TME is performed in patients with technically challenging rectal cancer, exposure, resection, and anastomosis will be more challenging, and the cooperation between the surgeon and the assistant will be severely tested. Specifically, performing the procedure under poor visualization or an unstable surgical area may increase the risk of rectal wall or vascular trauma (35). In contrast, R-TME provides greater autonomy and enables the performance of precise sharp dissection, which may shorten procedure time and reduce bleeding (13). We believe that despite these challenging surgical conditions, the implementation of R-TME in technically challenging rectal cancer patients has not been hindered.

This study has several limitations. First, this study was a retrospective analysis using a single institutional database, which may lead to patient selection bias. Second, our nomogram requires further validation in other independent patient cohorts. Thirdly, the proportion of patients receiving neoadjuvant therapy in this study is low; nevertheless, we achieved a good quality of TME. Nonetheless, the present study provides new insights into the precise evaluation of surgically difficult patients and the utilization of robotics in mid-low rectal cancer.

## 5 Conclusion

We successfully established a preoperative nomogram to predict the surgical difficulty of L-TME. Furthermore, this study also indicated that R-TME has potential benefits for patients with technically challenging rectal cancer.

## Ethics approval

All the patients personally signed the consent. This study was conducted in accordance with the ethical principles outlined in the 1964 Declaration of Helsinki. Ethical approval for this study was obtained from the Institutional Review Board Ethics Committee at The Second Affiliated Hospital of Dalian Medical University.

## Data availability statement

The original contributions presented in the study are included in the article/**Supplementary Material**. Further inquiries can be directed to the corresponding authors.

## Ethics statement

This study was conducted in accordance with the ethical principles outlined in the 1964 Declaration of Helsinki. Ethical approval for this study was obtained from the Institutional Review Board Ethics Committee at The Second Affiliated Hospital of Dalian Medical University. The patients/participants provided their written informed consent to participate in this study.

## Author contributions

FG: Conceptualization and Writing – original draft. CX: Formal analysis, Writing – original draft. ZW: Visualization, Writing – original draft. RW: Data curation. JG: Methodology. YM: Data curation. JP: Conceptualization. QZ: Writing – review & editing. SR: Writing – review & editing.

## References

[1] Qu R Ma Y Tao L Bao X Zhou X Wang B. Features of colorectal cancer in China stratified by anatomic sites: A hospital-based study conducted in university-affiliated hospitals from 2014 to 2018. Chin J Cancer Res (2021) 33(4):500–11. doi: 10.21147/j.issn.1000-9604.2021.04.07 PMC8435820 34584375

[2] Wang Z Dan W Zhang N Fang J Yang Y. Colorectal cancer and gut microbiota studies in China. Gut Microbes (2023) 15(1):2236364. doi: 10.1080/19490976.2023.2236364 37482657 PMC10364665

[3] Dekker E Tanis PJ Vleugels JLA Kasi PM Wallace MB. Colorectal cancer. Lancet (2019) 394(10207):1467–80. doi: 10.1016/S0140-6736(19)32319-0 31631858

[4] Heald RJ Husband EM Ryall RD. The mesorectum in rectal cancer surgery–the clue to pelvic recurrence? Br J Surg (1982) 69(10):613–6. doi: 10.1002/bjs.1800691019 6751457

[5] Fleshman J Branda ME Sargent DJ Boller AM George VV Abbas MA. Disease-free survival and local recurrence for laparoscopic resection compared with open resection of stage II to III rectal cancer: follow-up results of the ACOSOG Z6051 randomized controlled trial. Ann Surg (2019) 269(4):589–95. doi: 10.1097/sla.0000000000003002 PMC6360134 30080730

[6] Chen K Cao G Chen B Wang M Xu X Cai W. Laparoscopic versus open surgery for rectal cancer: A meta-analysis of classic randomized controlled trials and high-quality Nonrandomized Studies in the last 5 years. Int J Surg (2017) 39:1–10. doi: 10.1016/j.ijsu.2016.12.123 28087370

[7] Roodbeen SX de Lacy FB van Dieren S Penna M Ris F Moran B. Predictive factors and risk model for positive circumferential resection margin rate after transanal total mesorectal excision in 2653 patients with rectal cancer. Ann Surg (2019) 270(5):884–91. doi: 10.1097/sla.0000000000003516 31634183

[8] Crippa J Grass F Dozois EJ Mathis KL Merchea A Colibaseanu DT. Robotic surgery for rectal cancer provides advantageous outcomes over laparoscopic approach: results from a large retrospective cohort. Ann Surg (2021) 274(6):e1218–e22. doi: 10.1097/sla.0000000000003805 32068552

[9] Kowalewski KF Seifert L Ali S Schmidt MW Seide S Haney C. Functional outcomes after laparoscopic versus robotic-assisted rectal resection: a systematic review and meta-analysis. Surg Endosc (2021) 35(1):81–95. doi: 10.1007/s00464-019-07361-1 32025924 PMC7746565

[10] Feroci F Vannucchi A Bianchi PP Cantafio S Garzi A Formisano G. Total mesorectal excision for mid and low rectal cancer: Laparoscopic vs robotic surgery. World J Gastroenterol (2016) 22(13):3602–10. doi: 10.3748/wjg.v22.i13.3602 PMC4814646 27053852

[11] Safiejko K Tarkowski R Koselak M Juchimiuk M Tarasik A Pruc M. Robotic-assisted vs. Standard laparoscopic surgery for rectal cancer resection: A systematic review and meta-analysis of 19,731 patients. Cancers (2021) 14(1):180. doi: 10.3390/cancers14010180 35008344 PMC8750860

[12] Jayne D Pigazzi A Marshall H Croft J Corrigan N Copeland J. Effect of robotic-assisted vs conventional laparoscopic surgery on risk of conversion to open laparotomy among patients undergoing resection for rectal cancer: the ROLARR randomized clinical trial. Jama (2017) 318(16):1569–80. doi: 10.1001/jama.2017.7219 PMC5818805 29067426

[13] Kim MJ Park SC Park JW Chang HJ Kim DY Nam BH. Robot-assisted versus laparoscopic surgery for rectal cancer: A phase II open label prospective randomized controlled trial. Ann Surg (2018) 267(2):243–51. doi: 10.1097/sla.0000000000002321 28549014

[14] Kojima T Hino H Shiomi A Kagawa H Yamaoka Y Manabe S. Comparison between robotic-assisted and laparoscopic sphincter-preserving operations for ultra-low rectal cancer. Ann Gastroenterological Surg (2022) 6(5):643–50. doi: 10.1002/ags3.12564 PMC9444857 36091301

[15] Shiomi A Kinugasa Y Yamaguchi T Kagawa H Yamakawa Y. Robot-assisted versus laparoscopic surgery for lower rectal cancer: the impact of visceral obesity on surgical outcomes. Int J Colorectal Dis (2016) 31(10):1701–10. doi: 10.1007/s00384-016-2653-z 27599703

[16] Pan J Wang B Feng Z Sun Z Xia C Zhang Q. Robotic versus laparoscopic total mesorectal excision for mid-low rectal cancer with difficult anatomical conditions. Asian J Surg (2022) 45(12):2725–32. doi: 10.1016/j.asjsur.2022.01.026 35168863

[17] Burghgraef TA Sikkenk DJ Verheijen PM Moumni ME Hompes R Consten ECJ. The learning curve of laparoscopic, robot-assisted and transanal total mesorectal excisions: a systematic review. Surg Endosc (2022) 36(9):6337–60. doi: 10.1007/s00464-022-09087-z PMC9402498 35697853

[18] Escal L Nougaret S Guiu B Bertrand MM de Forges H Tetreau R. MRI-based score to predict surgical difficulty in patients with rectal cancer. Br J Surg (2018) 105(1):140–6. doi: 10.1002/bjs.10642 29088504

[19] Chin EC Leung CK Yu DJ Yu AP Bernal JK Lai CW. Effects of one-year once-weekly high-intensity interval training on body adiposity and liver fat in adults with central obesity: Study protocol for a randomized controlled trial. J Exercise Sci Fitness (2022) 20(2):161–71. doi: 10.1016/j.jesf.2022.03.003 PMC8943247 35401766

[20] Yang Y Wang HY Chen YK Chen JJ Song C Gu J. Current status of surgical treatment of rectal cancer in China. Chin Med J (Engl) (2020) 133(22):2703–11. doi: 10.1097/CM9.0000000000001076 PMC7647505 32889914

[21] Li C Guan Z Zhao Y Sun T Li Z Wang W. Predictors of pathologic complete response in patients with residual flat mucosal lesions after neoadjuvant chemoradiotherapy for locally advanced rectal cancer. Chin J Cancer Res (2022) 34(4):383–94. doi: 10.21147/j.issn.1000-9604.2022.04.06 PMC9468014 36199540

[22] Keller DS Berho M Perez RO Wexner SD Chand M. The multidisciplinary management of rectal cancer. Nat Rev Gastroenterol Hepatol (2020) 17(7):414–29. doi: 10.1038/s41575-020-0275-y 32203400

[23] Ito M Sugito M Kobayashi A Nishizawa Y Tsunoda Y Saito N. Relationship between multiple numbers of stapler firings during rectal division and anastomotic leakage after laparoscopic rectal resection. Int J Colorectal Dis (2008) 23(7):703–7. doi: 10.1007/s00384-008-0470-8 18379795

[24] Balachandran VP Gonen M Smith JJ DeMatteo RP. Nomograms in oncology: more than meets the eye. Lancet Oncol (2015) 16(4):e173–80. doi: 10.1016/s1470-2045(14)71116-7 PMC4465353 25846097

[25] Yuan Y Tong D Liu M Lu H Shen F Shi X. An MRI-based pelvimetry nomogram for predicting surgical difficulty of transabdominal resection in patients with middle and low rectal cancer. Front Oncol (2022) 12:882300. doi: 10.3389/fonc.2022.882300 35957878 PMC9357897

[26] Yamaoka Y Yamaguchi T Kinugasa Y Shiomi A Kagawa H Yamakawa Y. Mesorectal fat area as a useful predictor of the difficulty of robotic-assisted laparoscopic total mesorectal excision for rectal cancer. Surg Endosc (2019) 33(2):557–66. doi: 10.1007/s00464-018-6331-9 30006838

[27] Yamamoto T Kawada K Kiyasu Y Itatani Y Mizuno R Hida K. Prediction of surgical difficulty in minimally invasive surgery for rectal cancer by use of MRI pelvimetry. BJS Open (2020) 4(4):666–77. doi: 10.1002/bjs5.50292 PMC7397373 32342670

[28] de'Angelis N Pigneur F Martínez-Pérez A Vitali GC Landi F Gómez-Abril SA. Assessing surgical difficulty in locally advanced mid-low rectal cancer: the accuracy of two MRI-based predictive scores. Colorectal Dis (2019) 21(3):277–86. doi: 10.1111/codi.14473 30428156

[29] Kim JY Kim YW Kim NK Hur H Lee K Min BS. Pelvic anatomy as a factor in laparoscopic rectal surgery: a prospective study. Surg Laparosc Endosc Percutaneous Techniques (2011) 21(5):334–9. doi: 10.1097/SLE.0b013e31822b0dcb 22002269

[30] Cai ZH Zhang Q Fu ZW Fingerhut A Tan JW Zang L. Magnetic resonance imaging-based deep learning model to predict multiple firings in double-stapled colorectal anastomosis. World J Gastroenterol (2023) 29(3):536–48. doi: 10.3748/wjg.v29.i3.536 PMC9850934 36688017

[31] Park SY Lee SM Park JS Kim HJ Choi GS. Robot Surgery Shows Similar Long-term Oncologic Outcomes as Laparoscopic Surgery for Mid/Lower Rectal Cancer but Is Beneficial to ypT3/4 After Preoperative Chemoradiation. Dis Colon Rectum (2021) 64(7):812–21. doi: 10.1097/dcr.0000000000001978 33833141

[32] Baek SJ Kim CH Cho MS Bae SU Hur H Min BS. Robotic surgery for rectal cancer can overcome difficulties associated with pelvic anatomy. Surg Endosc (2015) 29(6):1419–24. doi: 10.1007/s00464-014-3818-x 25159651

[33] Feng Q Yuan W Li T Tang B Jia B Zhou Y. Robotic versus laparoscopic surgery for middle and low rectal cancer (REAL): short-term outcomes of a multicentre randomised controlled trial. Lancet Gastroenterol Hepatol (2022) 7(11):991–1004. doi: 10.1016/s2468-1253(22)00248-5 36087608

[34] Silva-Velazco J Dietz DW Stocchi L Costedio M Gorgun E Kalady MF. Considering value in rectal cancer surgery: an analysis of costs and outcomes based on the open, laparoscopic, and robotic approach for proctectomy. Ann Surg (2017) 265(5):960–8. doi: 10.1097/sla.0000000000001815 27232247

[35] Numata M Kazama K Onodera A Hara K Atsumi Y Okamoto H. Short-term outcomes following robotic-assisted laparoscopic surgery for technically demanding rectal cancer. Anticancer Res (2020) 40(4):2337–42. doi: 10.21873/anticanres.14201 32234935

