# Supplementary material for: Nomogram for predicting the surgical difficulty of laparoscopic total mesorectal excision and exploring the technical advantages of robotic surgery
Source: Front Oncol. 2024 Jan 26;14:1303686. doi: 10.3389/fonc.2024.1303686 (PMC10860337; doi:10.3389/fonc.2024.1303686)
Supplement: Supplementary file 1 [file Table_1.docx]

| **Supplemental Table 1** Patient features between R-TME and L-TME in patients with technically challenging rectal cancer | | | |
| --- | --- | --- | --- |
|  | L-TME (N = 78) | R-TME (N = 57) | p |
| Sex [n (%)] |  |  | 0.478 |
| Male | 59 (75.6%) | 40 (70.2%) |  |
| Female | 19 (24.4%) | 17 (29.8%) |  |
| Age [median (IQR), years] | 66 (60.8-71.0) | 65 (57.5-71.5) | 0.527 |
| Hypertension [n (%)] | 19 (24.4%) | 18(31.6%) | 0.353 |
| Diabetes [n (%)] | 8(10.3%) | 5(8.8%) | 0.773 |
| Cardiac disease [n (%)] | 4 (5.1%) | 4 (7.0%) | 0.721 |
| Respiratory disease [n (%)] | 6 (7.7%) | 3 (5.3%) | 0.733 |
| ASA score [n (%)] |  |  | 0.818 |
| I | 48 (61.5%) | 32 (56.1%) |  |
| II | 25 (32.1%) | 21 (36.8%) |  |
| III | 5 (6.4%) | 4 (7.0%) |  |
| Smoking history [n (%)] | 34 (43.6%) | 17 (29.8%) | 0.103 |
| Drinking history [n (%)] | 17 (21.8%) | 11 (19.3%) | 0.724 |
| Prostate hypertrophy [n (%)] | 10 (12.8%) | 11 (19.3%) | 0.308 |
| Preoperative chemotherapy [n (%)] | 9 (11.5%) | 4 (7.0%) | 0.379 |
| Preoperative radiotherapy [n (%)] | 6 (7.7%) | 2 (3.5%) | 0.467 |
| Previous abdominal surgery [n (%)] | 11 (14.1%) | 7 (12.3%) | 0.758 |
| CEA abnormal [n (%)] | 23 (29.5%) | 11 (19.3%) | 0.178 |
| CA199 abnormal [n (%)] | 6 (7.7%) | 3 (5.3%) | 0.733 |
| Anemia [n (%)] | 11 (14.1%) | 4 (7.0%) | 0.196 |
| Hypoproteinemia [n (%)] | 7 (9.0%) | 4 (7.0%) | 0.760 |
| Tumor diameter [median (IQR), cm] | 5.1 (4.5-6.1) | 5 (4-5.7) | 0.078 |
| Tumor height [median (IQR), cm] | 6 (4.9-7.8) | 5.9 (4.1-8) | 0.888 |
| Tumor differentiation [n (%)] |  |  | 0.008 |
| Poor | 11 (14.1%) | 13 (22.8%) |  |
| Moderate | 63 (80.8%) | 33 (57.9%) |  |
| High | 4 (5.1%) | 11 (19.3%) |  |
| Pathological T stage [n (%)] |  |  | 0.824 |
| T1 | 8 (10.3%) | 5 (8.8%) |  |
| T2 | 20 (25.6%) | 14 (24.6%) |  |
| T3 | 36 (46.2%) | 24 (42.1%) |  |
| T4 | 14 (17.9%) | 14 (24.6%) |  |
| Pathological N stage [n (%)] |  |  | 0.750 |
| N0 | 51 (65.4%) | 34 (59.6%) |  |
| N1 | 13 (16.7%) | 10 (17.5%) |  |
| N2 | 14 (17.9%) | 13 (22.8%) |  |
| Tumor stage [n (%)] |  |  | 0.602 |
| I | 19 (24.4%) | 10 (17.5%) |  |
| II | 32 (41.0%) | 24 (42.1%) |  |
| III | 27 (34.6%) | 23 (40.4%) |  |
| BMI [mean ± SD, kg/m²] | 24.7 ± 3.3 | 25.5 ± 3.4 | 0.156 |
| MFA [[mean ± SD, cm2] | 24.0 ± 6.9 | 22.4 ± 7.5 | 0.288 |
| Interspinous distance [median (IQR), cm] | 95.3 (89.9-102.3) | 96.6 (89.5-107.9) | 0.146 |
| Inter-tuberous distance [median (IQR), cm] | 109.5 (102.1-114.3) | 113.0 (101.1-128.1) | 0.093 |
| Pelvic inlet [median (IQR), cm] | 115.9 (110.1-123.3) | 116.0 (108.8-123.9) | 0.954 |
| Pelvic outlet [median (IQR), cm] | 93.2 (85.4-101.1) | 93.3 (87.1-101.3) | 0.581 |
| Pelvic depth [median (IQR), cm] | 125.9 (119.6-137.5) | 123.5 (115.1-133.2) | 0.078 |

TME: Total mesorectal excision; ASA, American Society of Anesthesiologists Classification; BMI, body mass index; CEA: Carcinoembryonic antigen; CA 199: Carbohydrate antigen 199; TME: Total mesorectal excision; MFA: rectal mesenteric fat; IQR, interquartile range; SD, standard deviation.
